# Effect of Intolerance of Uncertainty and Resource Consumption on Therapeutic Strategies Chosen by Physiotherapists: Virtual Patient Study

**DOI:** 10.2196/73818

**Published:** 2025-11-18

**Authors:** Clémence Brun, Alexis Akinyemi, Laurène Houtin, Philippe Meidinger, Jimmy Antunes, Richard Monvoisin, Nicolas Pinsault, Oulmann Zerhouni

**Affiliations:** 1 AD-HOC Lab Bois-Colombes France; 2 Institut pour le Développement et l'Action en Sciences Sociales (IDASC) Bois-Colombes France; 3 Lorraine Laboratory for Psychology and Neuroscience Research on Behavioral Dynamics Nancy France; 4 CNRS, UMR 5525, VetAgro Sup, Grenoble INP, TIMC University of Grenoble Alpes Grenoble France; 5 University of Rouen Normandy, Centre de Recherche sur les Fonctionnements et Dysfonctionnements Psychologiques (CRFDP, EA 7475) Rouen France; 6 Center for Interdisciplinary Research in Rehabilitation and Social Integration, Faculty of Medicine, Université Laval Quebec City, QC Canada

**Keywords:** clinical reasoning, intolerance of uncertainty, physiotherapists, therapeutic strategy, simulation game

## Abstract

**Background:**

There is a growing interest in the influence of variables that may negatively influence the reasoning of health care professionals, namely intolerance of uncertainty, defined as health care professionals’ difficulty tolerating ambiguous situations that often trigger discomfort, overtesting, and suboptimal treatment choices, and resource consumption, defined as the ordering of unnecessary diagnostic tests that can waste resources and compromise decision‐making.

**Objective:**

Our purpose was to assess the influence of intolerance of uncertainty and resource consumption on the chosen therapeutic strategy.

**Methods:**

A total of 127 physiotherapists were challenged to play a simulation game and choose a therapeutic strategy for 3 cases of low back pain of increasing difficulty (easy, medium, and difficult). Their intolerance of uncertainty level and their resource consumption were measured (ie, requests for test results for each case).

**Results:**

Results showed that 87.4% (111/127) of participants chose the most appropriate strategy (ie, the one in line with the strategy recommended by experts) for the easy case, 46.5% (59/127) for the medium, and 29.1% (37/127) for the difficult. For the easy case, intolerance of uncertainty and resource consumption had a negative influence on the chosen therapeutic strategy, which was less appropriate (*β*=–4.03, *t*_121_=–3.92, *P*<.001). Moreover, resource consumption among highly intolerant individuals had a more adverse influence on the chosen strategy (*β*=–3.42, *t*_121_=–5.09, *P*<.001). Such results were not found for the medium and difficult cases.

**Conclusions:**

Resource consumption may have a negative influence in some cases on the treatment strategies chosen by the physiotherapists who are most intolerant of uncertainty. Future research avenues are suggested to ensure the quality of patient care concerning physiotherapists’ level of intolerance of uncertainty.

## Introduction

Deaths are extremely uncommon in specialties that offer non-nosological diagnoses (eg, nursing diagnostics and physiotherapeutic diagnoses), but mistakes frequently result in more or longer treatment. Three-quarters of these errors are cognitive and reasoning errors, and 80% of them might be prevented [1,2]. Health care professionals (HCPs) use clinical reasoning, which is defined as a “deliberate process of critical thinking about a clinical situation to reach a reasonable decision about an outcome, diagnosis, therapeutic action, or resolution of a particular patient problem” to arrive at a diagnosis and treatment proposal [3]. However, clinical reasoning is frequently performed without a thorough understanding of all the pertinent characteristics and in medical environments that are highly susceptible to uncertainty, which might result in diagnostic uncertainty [3-7]. Diagnostic uncertainty is the subjective impression that HCPs feel when they cannot offer a correct and clear explanation for the health problem described by their patients [8]. Suboptimal clinical reasoning and diagnostic uncertainty can be caused by a variety of variables and situational characteristics.

Among these, the overconfidence of HCPs has been the subject of much research [9-14]. Being confident in one’s diagnosis and patient management skills is not always a bad thing, especially because it can have a positive impact on how patients view their therapeutic relationship with their practitioner, especially if they are experiencing distress [15]. Instead, the issue is the disparity, if any, between practitioners’ confidence and the precision of their diagnosis, which might cause practitioners to overlook important information or particular therapy [14,16,17]. As a result, confidence in one’s choices is increasingly being investigated in health care settings, such as in vaccination choices, and needs to be further studied within the framework of clinical reasoning [18]. Authors such as Zwaan and Hautz [14] consider that the future in this field of research lies in the joint study of uncertainty tolerance in combination with overconfidence and diagnostic accuracy to determine where these calibration errors occur.

Given the pervasiveness of uncertainty in medical settings, a lack of uncertainty tolerance can negatively impact both patients (particularly when HCPs fail to express their doubt to them) and HCPs (eg, health and reasoning abilities) [3,5,6,19]. Overtesting and occasionally insufficient therapy prescriptions have direct effects on patients [3]. Therefore, a high degree of intolerance of uncertainty may directly affect the diagnosis and course of treatment that patients receive. This is suggested by Simmonds et al [7] in a study showing that physiotherapists with a high level of intolerance of uncertainty will tend to suggest to their patients with low back pain a slower and less active return to their physical and professional activities than professionals with a lower level of intolerance of uncertainty.

Physiotherapists are among HCPs likely to treat patients following a referral from another HCP (usually a general practitioner [GP]). However, they are increasingly required to treat patients as the first HCP they encounter as part of direct access to care, even as they are likely to treat a wide range of conditions, both of which create uncertainty [20,21]. They establish treatment goals and an appropriate rehabilitation program after conducting an evaluation of the patient’s strengths and weaknesses using the prescription [22,23]. Physiotherapists are often consulted for low back pain, a very common problem that most people will experience in their lives [24,25]. Although the symptoms are easily recognized, the cause is less so: only 15% of cases of low back pain are associated with pathology, such as a tumor, osteoporosis, or fracture, or with symptoms related to the nerve roots, whereas 85% of cases are classified as nonspecific [26]. Furthermore, a study by Fourré et al [27] showed that a low proportion of physiotherapists are aware of the guidelines for the management of low-back pain, and that this uncertainty leads to guideline-inconsistent behavior. The current literature on physiotherapists’ clinical reasoning, however, appears to be focused on the identification and classification of the steps of the reasoning process without any relation to the relevant resulting variables, namely the diagnosis thought to be the most likely for a given situation and, most importantly, the therapeutic strategy offered to the patient [22,28,29]. There are also few pedagogical tools designed for physiotherapists and even fewer tools for assessing their clinical reasoning [30]. Furthermore, these studies failed to consider the influence of contextual and physiotherapist-related characteristics, such as intolerance of uncertainty and confidence in one’s judgment, on clinical decision-making [7,31]. As a result, they disregarded how intolerance of uncertainty affected their investigation process and prescriptions, even though we know that HCPs who have a high threshold for intolerance of uncertainty frequently recommend further tests, which may be considered a waste of resources (ie, overtesting [3]).

This study aimed to assess whether physiotherapists propose therapeutic strategies to their patients aligned with strategies recommended by experts, which will subsequently be referred to as “most appropriate strategies” in the remainder of the paper [32]. This variable was chosen as the main dependent variable as most studies focus on measuring diagnostic accuracy of participants (ie, correctness of the final diagnosis [33]). While this measure has the advantage of being easy to measure numerically and to score objectively, authors such as Daniel et al ([34] as cited in [33]) stressed the importance of also measuring other elements that contribute to the assessment of diagnostic success, notably the use of additional diagnostic tests, or treatment decisions. By placing this study within the scope of therapeutic reasoning [35] and following the recommendations of Daniel et al [34], our goal was to assess the impact of physiotherapists’ intolerance of uncertainty on the therapeutic strategy they chose and determine whether resource consumption (akin to overtesting) interacts with intolerance of uncertainty to influence the strategy chosen. Second, a subgoal was to check whether physiotherapists exhibited a confidence calibration issue between the strategy offered and its appropriateness for the case (ie, similar to the calibration issues between diagnostics and their quality).

Four hypotheses were tested: (H1) a high level of intolerance of uncertainty and greater resources consumption is associated with the selection of less appropriate therapeutic strategies, (H2) a high level of intolerance of uncertainty is associated with greater consumption of resources (ie, more requests for test results), (H3) level of confidence in one’s therapeutic strategy will be negatively correlated with the selection of appropriate therapeutic strategies (ie, calibration issue), and (H4) intolerance of uncertainty will be positively associated with confidence in one’s diagnoses.

## Methods

### Study Design

The methodology of this study was inspired by the approaches used by Jette et al [36] and Keller et al [37], where physiotherapists were presented with clinical cases to propose appropriate therapeutic strategies and suspected diagnoses. In our study, a simulation game was used, tailored to meet specific objectives such as incorporating measures of intolerance of uncertainty and resource consumption, modifying case formats, and introducing varying levels of difficulty. The content of the cases was adapted from Meidinger et al [32], focusing on low back pain with or without serious spinal pathologies, a condition known for its diagnostic challenges [26].

### Participants

Participants were recruited via email and social media, using a convenience sampling method. An email was sent to a network of physiotherapists associated with a French university laboratory, outlining this study’s purpose, estimated participation time, and access details for the game. Participants were encouraged to share the email within their networks. Concurrently, similar information was posted on platforms such as Facebook (Meta), LinkedIn (Microsoft Corp), and Instagram (Meta). Private messages were also sent to physiotherapists on LinkedIn.

Overall, 287 physiotherapists were recruited, but 160 were excluded for not meeting inclusion criteria, resulting in a final sample of 127 participants (*M_a_*_ge_=33.70, *SD*_age_=8.94, 36 women, 71 men, 1 other, and 18 who did not specify their gender). Of these, 105 practiced in France, 2 in Belgium, and 1 in the Ivory Coast (*M*_Years experience_=10.30, *SD*_Years experience_=8.95, 22 did not provide this information).

### Procedure and Instruments

This study used a simulation game featuring 3 fictitious clinical cases, designed to assess clinical reasoning through interactive scenarios rather than traditional multiple-choice questions (Table 1; Multimedia Appendix 1). This choice aligns with the assertion by Halpern (1998, as cited in the scoping review of Berg et al [38]) that such questions often emphasize rote knowledge over critical thinking and reasoning. The simulation method used in this study allows for more ecological assessments, reflecting real-world clinical practice where critical thinking is essential [39]. The game began with an overview of its structure and rules, along with a reminder that advancing screens would prevent participants from returning, encouraging careful consideration before proceeding. On the next screen, participants’ level of intolerance of uncertainty when caring for their patients was measured using the IUS-12-H (Intolerance of Uncertainty Scale-12 Health Care Professionals) [40]. This scale, based on the IUS-12 (Intolerance of Uncertainty Scale-12), a generalist intolerance of uncertainty scale initially validated by Carleton et al [41], has been validated in a sample of HCPs and has shown good psychometric properties [40]. The IUS-12 is scored on a Likert scale ranging from “not at all characteristic of me” (1), “a little characteristic of me” (2), “somewhat characteristic of me” (3), “very characteristic of me” (4), to “entirely characteristic of me” (5).

**Table 1:** Names, visuals, and the type of game screen. It presents an overview of each screen encountered by participants in the simulation game, organized into 3 columns: name, screen, and screen type. The “name” column identifies each interface element by its purpose, such as introducing this study, presenting a clinical case, or collecting demographic information. The “screen” column specifies the format or layout used to display content—for example, whether clinical information was shown as a series of questions and answers. Finally, the “screen type” column indicates whether a screen was compulsory (displayed to all participants) or optional (available only if participants chose to access additional material, such as test results). See Multimedia Appendix 1 for a wider view of each screen.

Name	Screen	Screen type^a^
Game presentation screen	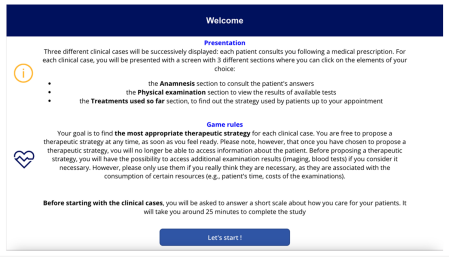	Compulsory
Intolerance of uncertainty scale screen	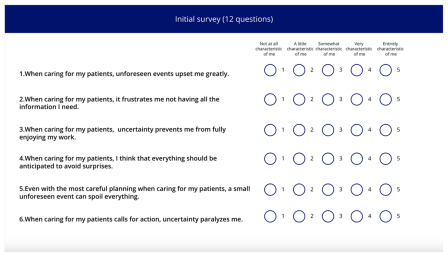	Compulsory
Clinical case screen with “question and answer” format	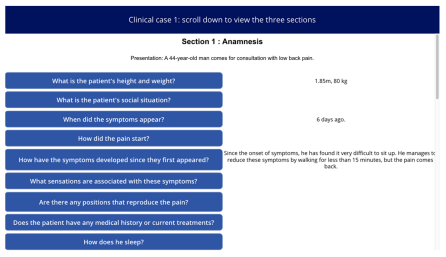	Compulsory
Therapeutic strategy selection screen	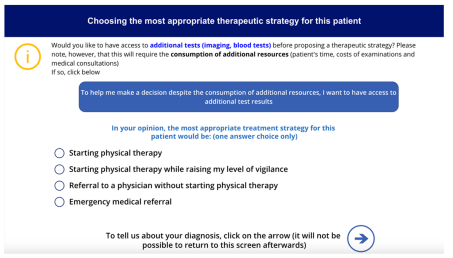	Compulsory
Additional test results selection screen	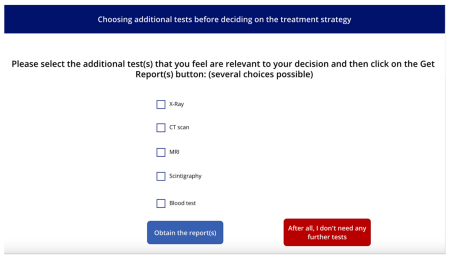	Optional
Additional test results screen	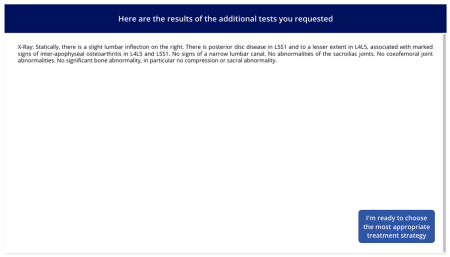	Optional
Suspected diagnosis and diagnosis confidence level screen	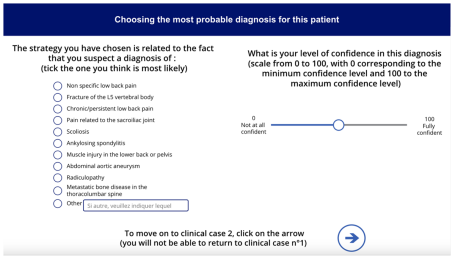	Compulsory
Case realism estimation screen	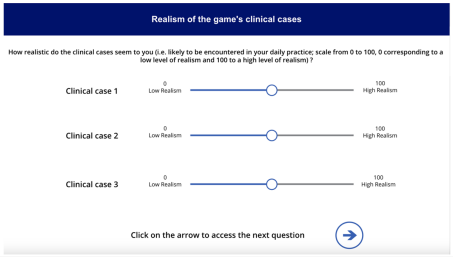	Compulsory
Case difficulty estimation screen	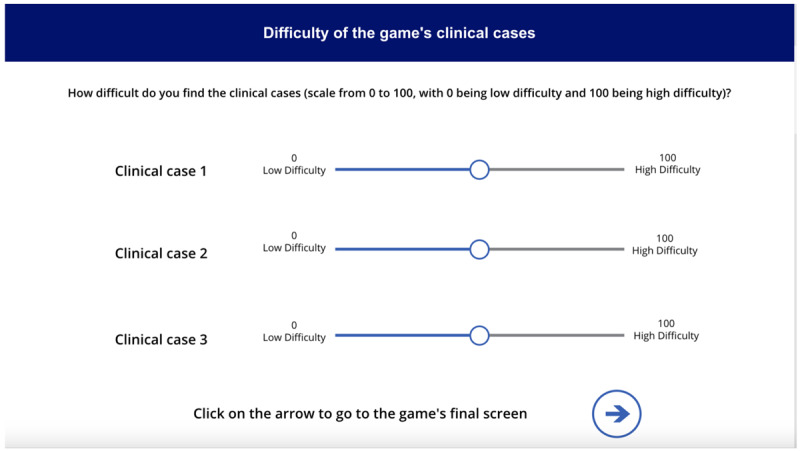	Compulsory
Sociodemographic data screen	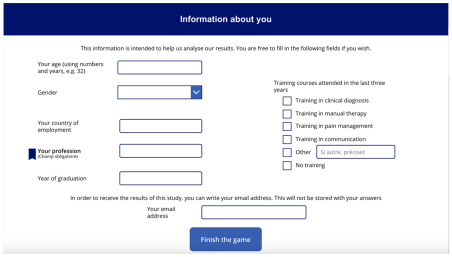	Compulsory

^a^Compulsory means the screen was displayed to all, optional means the screen was displayed only if selected.

The screen images shown in Table 1 can be viewed in full size in Multimedia Appendix 1. Each of the 3 featured cases was introduced successively, with each 1 presented in the same format: a brief description followed by a series of questions. The cases were adapted from Meidinger et al [32] and underwent a Delphi assessment to ensure varying levels of difficulty and to address the lack of studies on the reasoning process across different situational levels [42]. The cases were categorized into “easy,” “medium,” and “difficult,” reflecting varying degrees of diagnostic clarity and complexity: the “easy” case suggested a single, highly probable diagnosis (eg, “99% chance it’s Diagnostic 1”), the “medium” case involved a couple of competing diagnoses with differing probabilities (eg, “30% for Diagnostic 1, 70% for Diagnostic 2”), and the “difficult” case required evaluating several potential diagnoses with similar likelihoods (eg, “20% for Diagnostic 1, 30% for Diagnostic 2, 40% for Diagnostic 3, and 10% for Diagnostic 4”). These levels were designed to align with the levels of difficulty identified by physiotherapists in the study by Josephson et al [26] on low back pain. Clinical cases were presented as questions to be asked (eg, “What is the patient’s height and weight? 1.85 m, 80 kg”), reflecting medical practice where patient details are revealed through inquiry, encouraging multifaceted thinking [43]. The clinical cases were reviewed for consistency by a physiotherapist and a GP, with radiologists consulted on specific details. After implementation in a digital tool, they underwent a second evaluation by 4 independent physiotherapists using the same criteria. Full cases are available in the Multimedia Appendix 2.

Following the initial validation, the clinical cases were implemented in Microsoft PowerApps, a platform for developing tailored applications. They were presented in the order of “easy,” “difficult,” and “medium,” following reviewer feedback to enhance engagement and reduce decision-making bias. Each case began with essential patient details (eg, “a 27-year-old male comes in for low back pain”), similar to what patients typically share at the start of an appointment, followed by a list of questions categorized into anamnesis, physical examination, and treatment options. Participants could view answers in real-time, simulating a clinical report-writing process (see screen “clinical case screen with 'question or answer' format” in Table 1).

After each case, subsequent screens prompted participants to select the most appropriate intervention from predefined options before moving on to the next case. The scoring grid for treatment strategies was developed based on expert consensus, distinguishing between initiating treatment and maintaining vigilance (Table 2). Participants could access additional test results (ie, x-ray, computed tomography scan, magnetic resonance imaging, scintigraphy, and blood tests) via a dedicated screen, with reminders on judicious use to avoid unrealistic overuse. A button allowed them to return to the treatment strategy screen, though accessing the results screen was recorded. On the next screen, participants selected their suspected diagnosis from a predefined list based on scientific literature and the *ICD-11* (*International Statistical Classification of Diseases, Eleventh Revision*) [32,44,45], with an “other” option allowing written input. They also rated their confidence in the diagnosis on a 0-100 scale, from “not at all confident” to “fully confident.” Following the clinical cases, participants rated the realism and difficulty of each case on scales from 0 (“low realism or difficulty”) to 100 (“high realism or difficulty”). These ratings were assessed after all 3 cases to provide participants with reference points for comparison, while minimizing the influence of any single case on the evaluation of the others.

Finally, participants were invited to share sociodemographic data by completing blank fields, including age, profession (ie, to confirm they were physiotherapists), country of practice (ie, to explore potential differences across regions), graduation year (ie, to approximate experience level), and gender (ie, via a drop-down menu with “male,” “female,” “do not wish to answer,” and “other” and a custom field). They could also optionally share their email address.

**Table 2:** Scoring grid of strategies for each case^a^.

	Easy case	Medium case	Difficult case
Beginning of physiotherapy treatment	2	0	0
Beginning physiotherapy treatment while maintaining a high level of vigilance	1	2	1
Referral to a physician without starting physiotherapy treatment	0	1	2
Emergency medical referral	–1	–1	–1

^a^Scoring was based on the notion of distance from a correct answer. A score of 2 was assigned to the strategy considered to be the most appropriate for the case presented. A score of 1 was assigned to the strategy considered to be good but not optimal. A score of 0 was assigned to the strategy considered to be neutral in that it has no harmful effect but no real positive effect. A score of –1 was assigned to the strategy considered to be contraindicated. This grid, based partly on the views of the Delphi experts, was devised by 3 physiotherapists by joint agreement.

### Statistical Analysis

First, we performed our main statistical analyses (1) on the therapeutic strategy chosen by HCPs to assess their suitability for each case and identify factors influencing HCPs’ choices (ie, with a frequency table and linear regression model). The goal was to confirm that most participants chose the correct strategy and assess the impact of their intolerance of uncertainty level and resource use on their choices. Then, (2) regression analyses were performed to see whether physiotherapists’ level of intolerance of uncertainty influenced their resource consumption. Then, correlational analyses were conducted to determine whether physiotherapists’ confidence was related to (3) the therapeutic strategy they chose and (4) to their level of intolerance of uncertainty.

For the secondary analyses, (5) frequency analyses were performed on participants’ confidence in their choices, level of realism attributed to the cases, and level of difficulty attributed to each case. The aim was to quantify participants’ level of confidence, ensure cases were rated realistically (ie, above 70 on the provided scale), and confirm increasing difficulty across cases.

Finally, we conducted exploratory analyses by performing frequency analyses on the diagnoses selected by participants for each case. These analyses aimed to confirm that most participants identified the correct diagnosis and to ascertain that the number of selected diagnoses increased with case difficulty (as suggested in the “procedure and instruments” section).

### Ethical Considerations

Ethical standards were upheld throughout the recruitment process, adhering to national and institutional guidelines, including the Helsinki Declaration. The CERGA (ie, Comité d’Éthique pour la Recherche de l’Université Grenoble Alpes) reviewed and approved this study without requiring additional precautions. A confirmation letter from the CERGA stating this is available from the authors upon request. Data collected from participants were anonymized, ensuring confidentiality, and participation was voluntary, with explicit consent obtained through a simple yes or no question regarding their willingness to participate (“Do you give your consent to participate in this study?”). There was no financial compensation for participation.

## Results

### Assessment of the Therapeutic Strategies Chosen and the Factors Influencing This Choice

**Figure 1:**
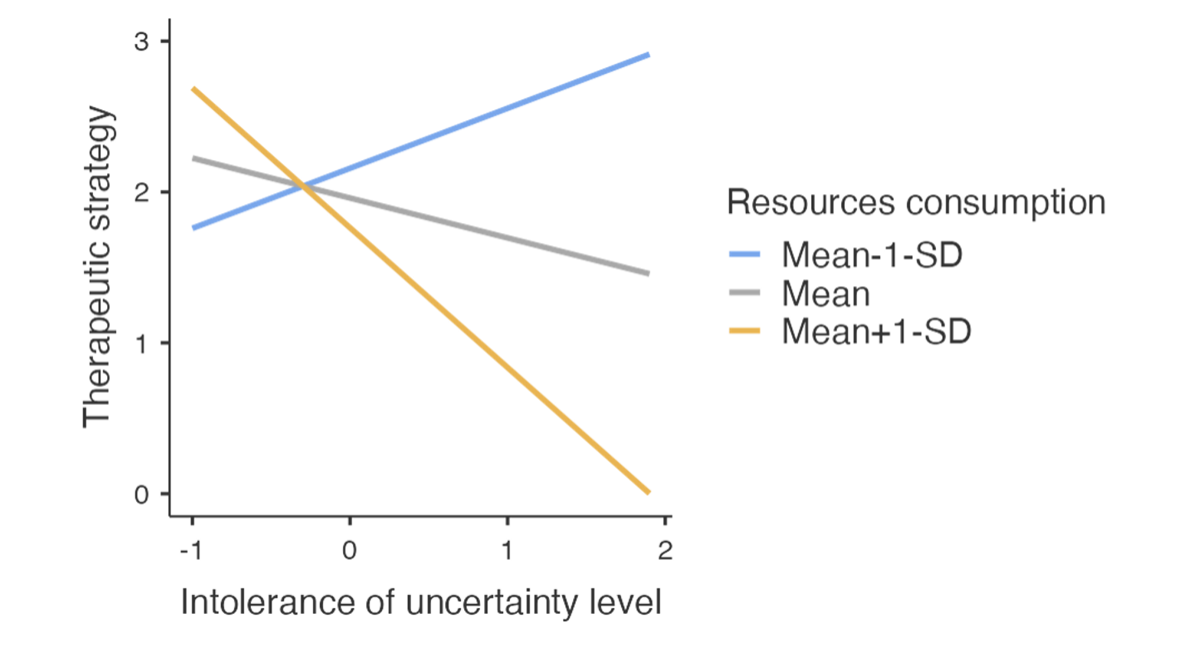
Quality of therapeutic strategy chosen by level of intolerance of uncertainty and resource consumption. Uncertainty-tolerant physiotherapists (mean-1-SD, on the left of the graph) propose more appropriate therapeutic strategies (ie, in line with expert recommendations) when they consume a high level of resources (orange line) than when they consume an average amount (gray line), and even more so than when they consume a low level of resources (blue line). For physiotherapists with an average level of intolerance of uncertainty (mean, on the center of the graph), the chosen therapeutic strategy becomes increasingly appropriate as resource consumption decreases—that is, those consuming few resources select more appropriate strategies than those consuming an average amount, who in turn select more appropriate strategies than those consuming many resources. This pattern is most pronounced in uncertainty-intolerant physiotherapists (mean+1-SD, on the right of the graph), for whom low resource consumption leads to substantially more appropriate strategies than average consumption, which itself yields more appropriate strategies than high consumption.

In the easy case analysis, 87.4% (111/127) of participants chose the most appropriate strategy (ie, beginning of physiotherapy treatment), 11.8% (15/127) chose the good but suboptimal strategy (ie, beginning physiotherapy treatment while maintaining a high level of vigilance), and 0.8% (1/127) chose the neutral strategy (ie, referral to a physician without starting physiotherapy treatment). No participants chose the contraindicated strategy (ie, emergency medical referral). Most participants did not use resources, with only 3.9% (5/127) requesting test results. Regression analysis revealed a significant negative effect of HCPs’ intolerance of uncertainty on their selected therapeutic strategy (*β*=–0.26, *t*_121_=–4.08, *P*<.001) and a significant negative effect of resource use on the selected strategy (*β*=–4.03, *t*_121_=–3.92, *P*<.001). Specifically, resource consumption positively influenced the quality of therapeutic strategies for uncertainty-tolerant individuals (mean-1-SD, *β*=1.02, *t*_121_=1.96, *P*=.053), but negatively impacted those with moderate (mean, *β*=–1.20, *t*_121_=–5.91, *P*<.001), and low tolerance of uncertainty (mean+1-SD, *β*=–3.42, *t*_121_=–5.09, *P*<.001, cf. Figure 1). Similarly, intolerance of uncertainty improved strategies when few resources were consumed (mean-1-SD), *β*=0.40, *t*_121_=2.59, *P*=.011), but reduced quality with average (mean, *β*=–0.26, *t*_121_=–4.08, *P*<.001), or high resource use (mean+1-SD, *β*=–0.93, *t*_121_=–4.53, *P*<.001, cf. Figure 1). The model also included age, experience, and gender; however, none significantly influenced the chosen strategy (*P*_age_=.90, *P*_experience_=.73, and *P*_gender_=.80).

For the medium case, 46.5% (59/127) of participants chose the most appropriate strategy (ie, beginning physiotherapy treatment while maintaining a high level of vigilance), 28.3% (36/127) chose the neutral strategy (ie, beginning of physiotherapy treatment), and 25.2% (32/127) chose the good but not optimal strategy (ie, referral to a physician without starting physiotherapy treatment). The contraindicated strategy (ie, emergency medical referral) was not chosen by any participant. Just under half of the participants (54/127, 42.5%) requested test results. Regression analysis showed no significant effect of intolerance of uncertainty on the chosen strategy (*β*=–0.03, *t*_121_=–0.19, *P*=.85), nor did age, experience, gender, or resource use significantly affect the chosen strategy (*P*_age_=.74, *P*_experience_=.70, *P*_gender_=.72, and *P*_ressources consumption_=.03).

In the difficult case, 41.7% (53/127) of participants chose the good but not optimal strategy for the case (ie, beginning physiotherapy treatment while maintaining a high level of vigilance), while 29.1% (37/127) chose the most appropriate strategy (ie, referral to a physician without starting physiotherapy treatment), 18.9% (24/127) chose the contraindicated strategy (ie, emergency medical referral), and 10.2% (13/127) chose the neutral strategy (ie, beginning of physiotherapy treatment). Resource use was again notable, with 44.1% (56/127) requesting test results. Regression analysis showed no significant effect of intolerance of uncertainty on the chosen strategy (*β*=–0.01, *t*_121_=–0.71, *P*=.48), and other variables (ie, age, experience, gender, and resource use) did not significantly influence the chosen strategy (*P*_age_=.65, *P*_experience_=.60, *P*_gender_=.51, and *P*_ressources consumption_=.01).

### Assessment of the Resource Consumption and the Factors Influencing This Choice

For the easy case, only 3.9% (5/127) of participants consumed resources (ie, examination results), with no significant effect of intolerance of uncertainty on their tendency to consume resources (*β*=–0.02, *t*_122_=–0.56, *P*=.58), or other variables such as age, gender, and experience (*P*_age_=.48, *P*_experience_=.54, and *P*_gender_=.66). For the medium case, 42.5% (54/127) of participants consumed resources, similarly unaffected by intolerance of uncertainty (*β*=0.14, *t*_122_=1.55, *P*=.13) or age, gender, or experience (*P*_age_=.70, *P*_experience_=.64, and *P*_gender_=.39). As for the difficult case, 44.1% (56/127) consumed resources, again with no significant effect of intolerance of uncertainty (*β*=0.01, *t*_122_=0.06, *P*=.95), or other age, gender, and experience (*P*_age_=.06, *P*_experience_=.04, and *P*_gender_=.57).

### Assessments of the Relation Between Confidence and Therapeutic Strategy and Confidence and Intolerance of Uncertainty

Participants’ confidence in their decisions was positively correlated with the quality of the therapeutic strategy chosen for the easy case (*r*=.41, *P*<.001), but negatively correlated for the medium (*r*=–0.31, *P*<.001) and difficult cases (*r*=–0.39, *P*<.001). Intolerance of uncertainty did not significantly correlate with confidence in decisions across all cases (easy: *r*=–0.08, *P*=.40; medium: *r*=–0.15, *P*=.09; and difficult: *r*=–0.09, *P*=.32).

### Assessment of Diagnostic Confidence, Realism, and Difficulty for Each Case

Participants rated their confidence at an average of 75.7 (SD=17.16) for the easy case, 61.9 (SD=19.88) for the medium case, and 62.6 (SD=24.04) for the difficult case. The realism attributed to the cases averaged 86.5 (SD=18.64) for the easy case, 82.9 (SD=21.32) for the medium case, and 80.0 (SD=24.72) for the difficult case. Finally, difficulty levels were rated at 34.7 (SD=23.45) for the easy case, 48.8 (SD=23.81) for the medium case, and 55.0 (SD=24.22) for the difficult case.

### Diagnostic Selected for Each Case

Diagnoses selected by participants for each case are shown in Table 3.

**Table 3:** Percentage of diagnoses selected for each case.

Diagnostic	Easy case	Medium case	Difficult case
Nonspecific low back pain (%)	120 (94.5)	8 (6.3)	23 (18.1)
Fracture of the L5^a^ vertebral body (%)	0 (0)	0 (0)	4 (3.1)
Chronic or persistent lumbago (%)	4 (3.1)	21 (16.5)	12 (9.4)
Pain related to the sacroiliac joint (%)	0 (0)	27 (21.3)	11 (8.7)
Scoliosis (%)	0 (0)	9 (7.1)	0 (0)
Ankylosing spondylitis (%)	0 (0)	56 (44.1)	4 (3.1)
Muscle injury in the lower back or pelvis (%)	0 (0)	1 (0.8)	4 (3.1)
Abdominal aortic aneurysm (%)	2 (1.6)	0 (0)	1 (0.8)
Radiculopathy (%)	0 (0)	0 (0)	8 (6.3)
Metastatic bone disease in the thoracolumbar spine (%)	0 (0)	0 (0)	53 (41.7)
Other (%)	1 (0.8)	5 (3.9)	7 (5.5)

^a^L5: fifth and final vertebra in the lumbar spine.

## Discussion

### Summary of Results

Improving clinical reasoning requires understanding how factors such as intolerance of uncertainty influence therapeutic strategy selection. In this study, 127 physiotherapists evaluated 3 clinical cases of increasing difficulty and chose therapeutic strategies. We measured participants’ intolerance of uncertainty and their consumption of resources (eg, additional test results) to assess how these variables affected their reasoning and strategy recommendations.

For the easy and medium cases, 87.4% (111/127) and 46.5% (59/127) of physiotherapists, respectively, selected the most appropriate strategy. In contrast, for the difficult case, 41.7% (53/127) chose a strategy deemed good but suboptimal, and participants considered a greater number of possible approaches. Regression analyses indicated that intolerance of uncertainty and resource consumption were significantly associated with selecting less appropriate strategies in the easy case (supporting hypothesis H1 only for that scenario), while no such associations appeared for the medium or difficult cases. Notably, high intolerance of uncertainty seemed to negatively impact strategy choice when resources were used, raising questions about the value of testing for these individuals, given its detrimental effect on treatment decisions and societal cost. Age, gender, and experience did not significantly affect strategy choice or resource consumption, consistent with the findings of Aron et al [46]. Regression models also showed no significant effect of intolerance of uncertainty on resource use across any case, failing to support hypothesis H2; this may reflect the French context, where physiotherapists cannot independently prescribe tests. Future investigations in systems granting prescribing rights (eg, Canada or Australia) or in France if regulations change [47] could clarify this relationship. For hypothesis H3, a negative correlation was found between the quality of the therapeutic strategy and the physiotherapist’s confidence for medium (*r*=–0.31) and difficult cases (*r*=–0.39), indicating that physiotherapists remained confident despite not choosing the most appropriate strategies. This suggests a calibration problem, consistent with the literature on overconfidence among HCPs, although it relates here to therapeutic strategy rather than diagnosis. Conversely, for the easy case, a positive correlation between confidence and strategy quality likely reflects that most participants correctly identified the simple scenario, yielding good calibration. Hypothesis H4, which posited a positive relationship between intolerance of uncertainty and diagnostic confidence (anticipating that those less tolerant might prematurely close on a diagnosis and thus report higher confidence [13,48]), was not supported. This result may reflect sampling bias, as the study’s demands likely attracted only the most motivated and confident participants, possibly skewing the findings. Future research could address this by including larger, more representative samples.

Secondary analyses revealed that participants perceived increasing difficulty across cases (34.7 for easy, 48.8 for medium, and 55.0 for difficult cases), with realism ratings above 80 for all cases (86.5, 82.9, and 80.0, respectively), suggesting reasonable validity for the cases designed in this study. Confidence levels decreased from 75.7 for the easy case to 61.9 for the medium case and slightly rebounded to 62.6 for the difficult case, echoing Staal et al [49]. This pattern aligns with findings from Haber et al [11] regarding confidence distribution in clinical decision-making (ie, 5% of their sample was “very confident,” 47% “confident,” 42% “somewhat confident,” 5% “not very confident,” and 0% “not at all confident”). The unexpected dip in confidence for the medium case—despite its intermediate complexity—may reflect participants’ lesser familiarity with its clinical presentation, a point we return to in the discussion.

Finally, exploratory analyses of diagnoses showed that most participants identified the correct diagnosis for each case, supporting the premise of the methodology that more diagnoses would be considered as case difficulty increased. Correct diagnoses fell from 94.5% (120/127) in the easy case to 44.1% (56/127) for the medium and 41.7% (53/127) for the difficult case, mirroring the confidence trend and underscoring the challenges of less familiar clinical scenarios.

### Do Most Physiotherapists Recommend Good Therapeutic Strategies?

To contextualize our findings, we compare them with Jette et al [36], who reported correct therapeutic strategy rates averaging 87% for musculoskeletal conditions, 88% for noncritical medical issues, and 79% for critical medical conditions, and with Keller et al [37], who found a 71.2% rate of correct management decisions. In our sample, physiotherapists achieved 87.4% for the easy case—comparable to Jette et al [36]—but only 46.5% and 29.1% for medium and difficult cases, respectively. However, when combining the most appropriate strategies with those labeled “good but not optimal,” success rates rose to 99.2% for the easy case, 71.7% for the medium case, and 70.6% for the difficult case, indicating that the sample was able to suggest strategies that were at least partially appropriate. Thus, while building on the work of Jette et al [36] and Keller et al [37], whose vignettes were concise and facilitated pattern recognition, this study contributes to the literature by offering scenarios that incorporated greater realism and complexity, including misleading information that required active information-seeking and critical evaluation. The high performance on the easy case underscores that familiar scenarios similar to those of Jette et al [36] primarily engage pattern recognition—a point explored further in the next section.

Despite these encouraging findings, additional benchmarks are needed to strengthen our conclusions. Forthcoming data from Lackenbauer et al [50] may offer further context, and future work should assess whether GPs and physiotherapists—who exhibit similar diagnostic accuracy [37]—differ in therapeutic recommendations. Although physiotherapists achieved relatively good rates when combining the most appropriate and good but not optimal strategies in our study, the selection of the most appropriate strategy decreased with increasing case difficulty. In addition, the medium and difficult cases yielded similar overall results, leading to hypotheses regarding the different treatment of medium and difficult cases compared to easy cases.

### Hypotheses on the Differences Observed Between Cases

The 3 explanatory hypotheses warrant exploration in future research to clarify the observed differences between cases. The first hypothesis is based on system 1/system 2 theory by Kahneman [4,10,51-54], which posits that rapid, heuristic-based system 1 reasoning is effective when HCPs recognize familiar patterns, whereas slow, analytic system 2 reasoning is deployed for analytical thinking. The easy case likely activated system 1, enabling rapid identification of a common low back pain presentation [1]. Conversely, the medium (ie, ankylosing spondylitis) and difficult (ie, metastatic bone disease) cases were rarer in primary care [45,55], requiring analytic engagement (system 2). We hypothesize that the activated heuristics in system 1 made HCPs susceptible to external influences—namely, intolerance of uncertainty and resource use—whereas system 2’s more deliberate processing in less familiar scenarios attenuated these effects [1]. Further research is needed to explore how these factors affect therapeutic strategies for less familiar clinical scenarios.

The second hypothesis relates to the concept of familiarity and the scope of practice of physiotherapists. Keller et al [37] found that specialization and exposure to similar cases increased recognition and the likelihood of suggesting appropriate therapeutic strategies. The simple case exemplifies this and may explain the high success rates observed. As such, a lack of familiarity with certain cases may exacerbate the effects of a physiotherapist’s specialization. We did not collect data on participants’ subspecialties (eg, pediatrics and maxillofacial), which may have influenced responses, particularly for cases outside their routine scope. Specialization should be controlled for in future studies, as it may have influenced physiotherapists’ responses in difficult cases, reinforcing system 2 use.

Finally, the third hypothesis focuses on physiotherapists’ perceptions of uncertainty. Although we did not directly measure perceived uncertainty, it can be hypothesized that the effects of intolerance of uncertainty and resource use were more pronounced in the easy case due to physiotherapists’ engagement, while the medium and difficult cases, being less familiar, may have induced a higher perception of uncertainty, leading to disengagement. Previous research indicates that intolerance of uncertainty, more than perceived uncertainty, drives health care decisions [18]. However, perceived uncertainty might cause physiotherapists to disengage, leading to fewer optimal treatment choices and dampening the effects of intolerance of uncertainty and resource use. Future work should investigate how perceived uncertainty affects strategy quality, as it may reflect physiotherapists’ engagement.

### Limitations

Several limitations should guide future work. First, the generalizability of our findings is limited: different HCPs interpret and reason about clinical cases in discipline-specific ways [56], so the impact of intolerance of uncertainty on decision-making among other HCPs may differ, which questions the applicability of the results to other HCPs and warrants further studies. Second, although participants were made aware of resource usage without explicit restraint, we cannot conclusively determine how the instructions influenced their decision to request additional tests. Future studies could vary instructions regarding access to patient information to evaluate how these constraints affect clinical reasoning and strategy selection.

Regarding the methodology used, although we carefully implemented the clinical cases using a relevant tool, several limitations of this format must be acknowledged. First, participants could not revisit earlier choices, which likely heightened their focus on patient data compared to real practice. Future studies should vary these instructions to see how they affect consultation outcomes. Second, the on-screen presentation, which eliminated the need to memorize or record patient details, may have simplified the task. Using drop-down menus or incorporating audio and real imaging (eg, radiographs) would enhance ecological validity by mimicking real-world consultation dynamics [57]. Finally, although we included self-reported realism ratings, we did not undertake a formal validation of the clinical cases. Applying established frameworks—such as the validation grid by Downing [58] or the guidelines by Schmutz et al [59]—would strengthen the methodological rigor of case design.

### Possible Improvements to the Tool

This study serves as a first step in understanding physiotherapists’ clinical reasoning using digital consultation simulation tools. However, the assessment method could be enhanced by incorporating established frameworks for clinical reasoning evaluation. Daniel et al [34] identify 7 components—information gathering, hypothesis generation, problem representation, differential diagnosis, leading diagnosis, diagnostic justification, and management or treatment—that could be systematically measured and linked to therapeutic outcomes. Adapting the tool to capture these discrete elements would allow a more fine-grained analysis of participants’ reasoning processes.

Incidentally, some measures could be expanded, notably as the IUS-12, which is the gold standard for intolerance of uncertainty assessment, comes with specific limitations. It does not capture how individuals represent uncertainty, react to specific uncertain situations, face different types of uncertainty, or in which cognitive domains they are particularly intolerant [60]. Moreover, self-report instruments are subject to social desirability bias and limited introspective accuracy. Although our observed range of IUS-12 scores indicates adequate variance, future research should triangulate self-reports with physiological measures (eg, skin conductance during uncertain scenarios), peer or supervisor assessments, and cognitive tasks combined with computational modeling (as in Sandhu et al [60]) to separate different forms of uncertainty and provide a more granular understanding of how uncertainty processing affects clinical reasoning. Additionally, incorporating attention-check items or repeated measures would further improve the reliability of the assessment.

Finally, revision of the tool could also involve updating the clinical cases and adding features. Cases 2 and 3 produced similar outcomes on multiple variables, suggesting that adjustments to their content or structure may be necessary to elicit distinct reasoning patterns. Enhancing immersion through expanded anamnesis (to replicate the breadth of history-taking) and replacing text-based test results with actual images could challenge participants’ interpretive skills and yield more realistic cognitive demands. As interpretation depends on the specialist and the referral context [57], this change could alter HCP reasoning, increase game complexity, and affect perceived uncertainty. Furthermore, implementing a module for real-time tracking of diagnostic hypotheses—akin to the method by Pelaccia et al [61] of monitoring hypothesis evolution—would enable evaluation of how HCPs integrate new information and revise their strategies, linking hypothesis generation and diagnostic reasoning directly to therapeutic decisions and filling in this gap identified by Duong et al [35]. Implementing a similar module for therapeutic strategies could, for instance, help assess the influence of patient information or test results on the selected strategies.

### Practical Implications for Resource Consumption and Management of Intolerance of Uncertainty

As noted by Infante-Guedes et al [20], research on physiotherapists’ tolerance of clinical uncertainty is sparse, particularly regarding its impact on resource use and societal costs. Our findings suggest that HPCs with high intolerance of uncertainty may be prone to suboptimal resource use, especially in simple cases that rely on pattern recognition. Future studies should evaluate whether these effects extend to more analytically demanding scenarios or remain confined to familiar presentations. New studies on uncertainty tolerance and resource use should also differentiate effects by test, as HCPs with higher tolerance order fewer tests [62], and diagnosis probability adjustments vary by test [63]. Interventions that display the cost of diagnostic tests—such as a gamified approach by Ishizuka et al [64]—could heighten cost awareness and reduce test overuse.

Looking ahead, as the direct access paradigm expands in France [25,46,65,66], physiotherapists will increasingly make independent diagnostic and therapeutic decisions, directly influencing patient pathways and health care expenditures. Our results imply that intolerance of uncertainty may detrimentally affect treatment choices—particularly regarding additional testing—in trained physiotherapists. Further research should develop strategies to mitigate this effect, potentially through targeted training in uncertainty management, decision-making frameworks that emphasize cost-effectiveness, and feedback systems that highlight calibration between confidence and accuracy. Together, these strategies could improve the quality of care provided and ensure that physiotherapists are prepared to make informed decisions in an evolving health care landscape.

The Multimedia Appendix 2 includes the 3 clinical cases of increasing difficulty used in the methodology presented in this paper. The document “clinical case 1 (difficulty level: easy - suggestive of common non-specific lumbago)” presents the first case, the document “clinical case 2 (difficulty level: medium - suggestive of ankylosing spondylitis)” presents the second case and the document “clinical case 3 (difficulty level: difficult - suggestive of metastatic bone disease in the thoracolumbar spine)” presents the third case.

## Appendix

Multimedia Appendix 1 SVG images of the pictures in Table 1.

Multimedia Appendix 2 Clinical cases.

## Abbreviations

CT: computed tomography
GP: general practitioner
HCP: health care professional
ICD-11: International Statistical Classification of Diseases, Eleventh Revision
IUS-12: Intolerance of Uncertainty Scale-12
IUS-12-H: Intolerance of Uncertainty Scale-12 Health Care Professionals
