# Supplementary figures and images for: Effect of Intolerance of Uncertainty and Resource Consumption on Therapeutic Strategies Chosen by Physiotherapists: Virtual Patient Study
Source: JMIR Rehabil Assist Technol. 2025 Nov 18;12:e73818. doi: 10.2196/73818 (PMC12673305; doi:10.2196/73818)

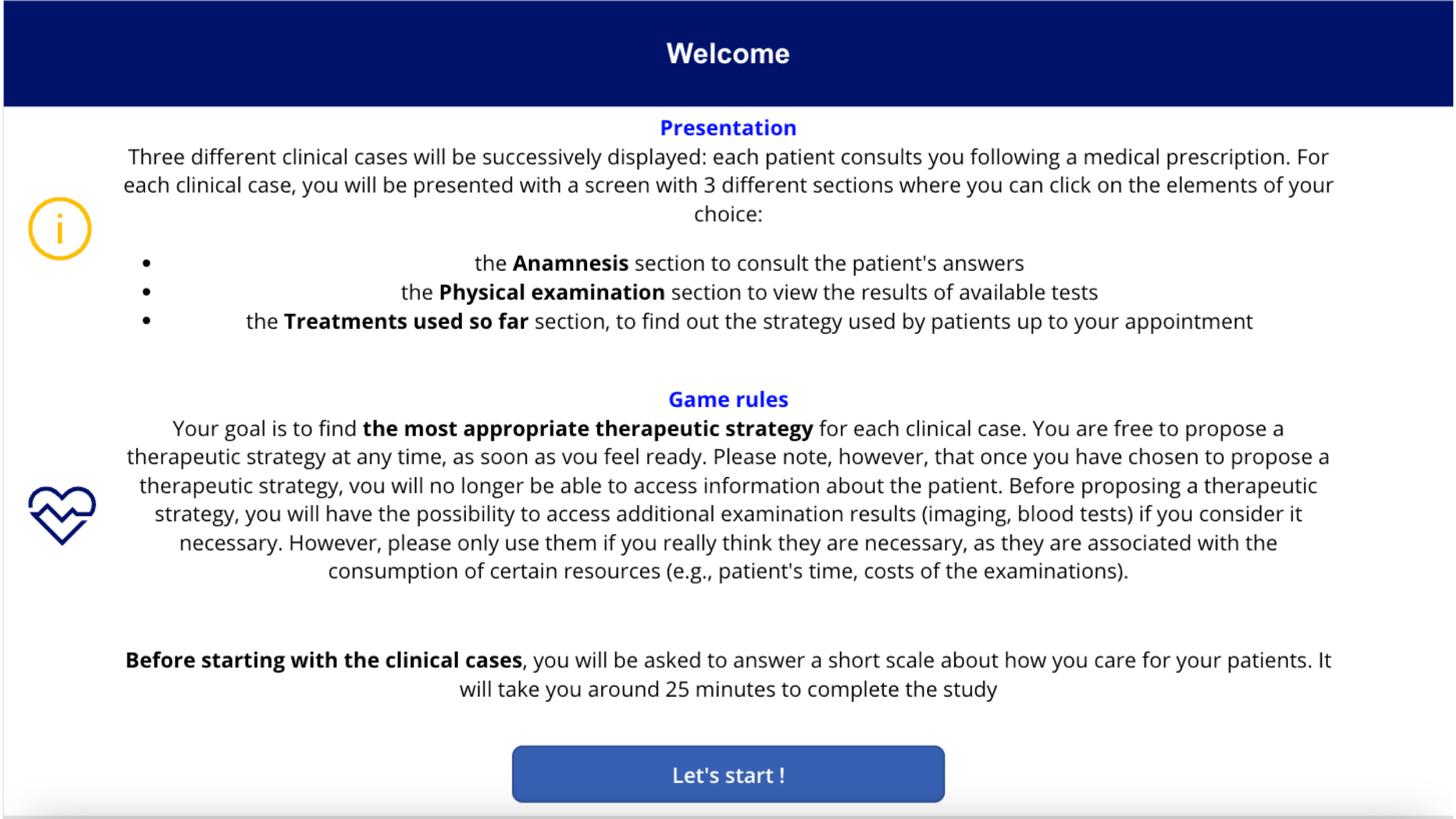

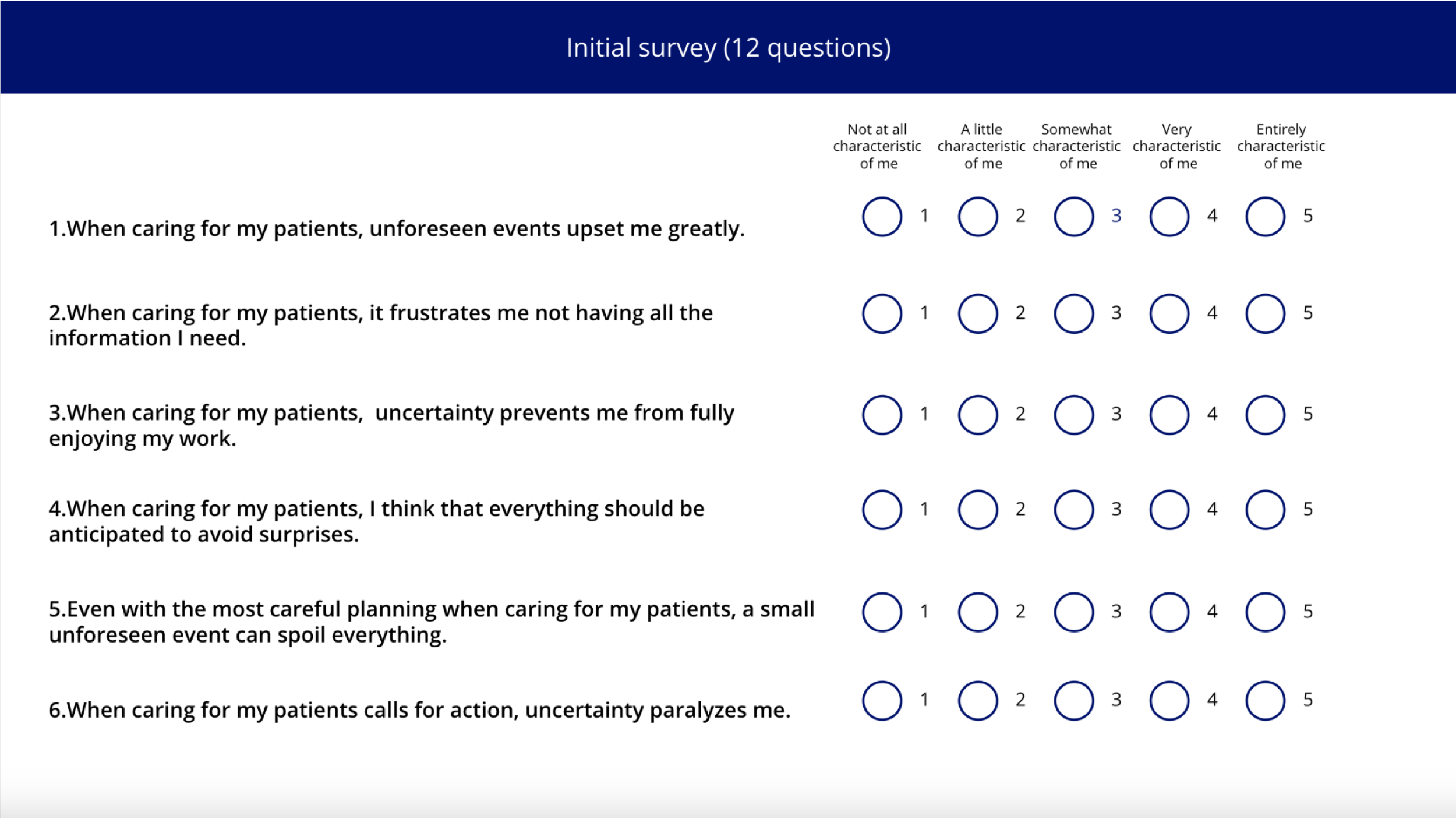

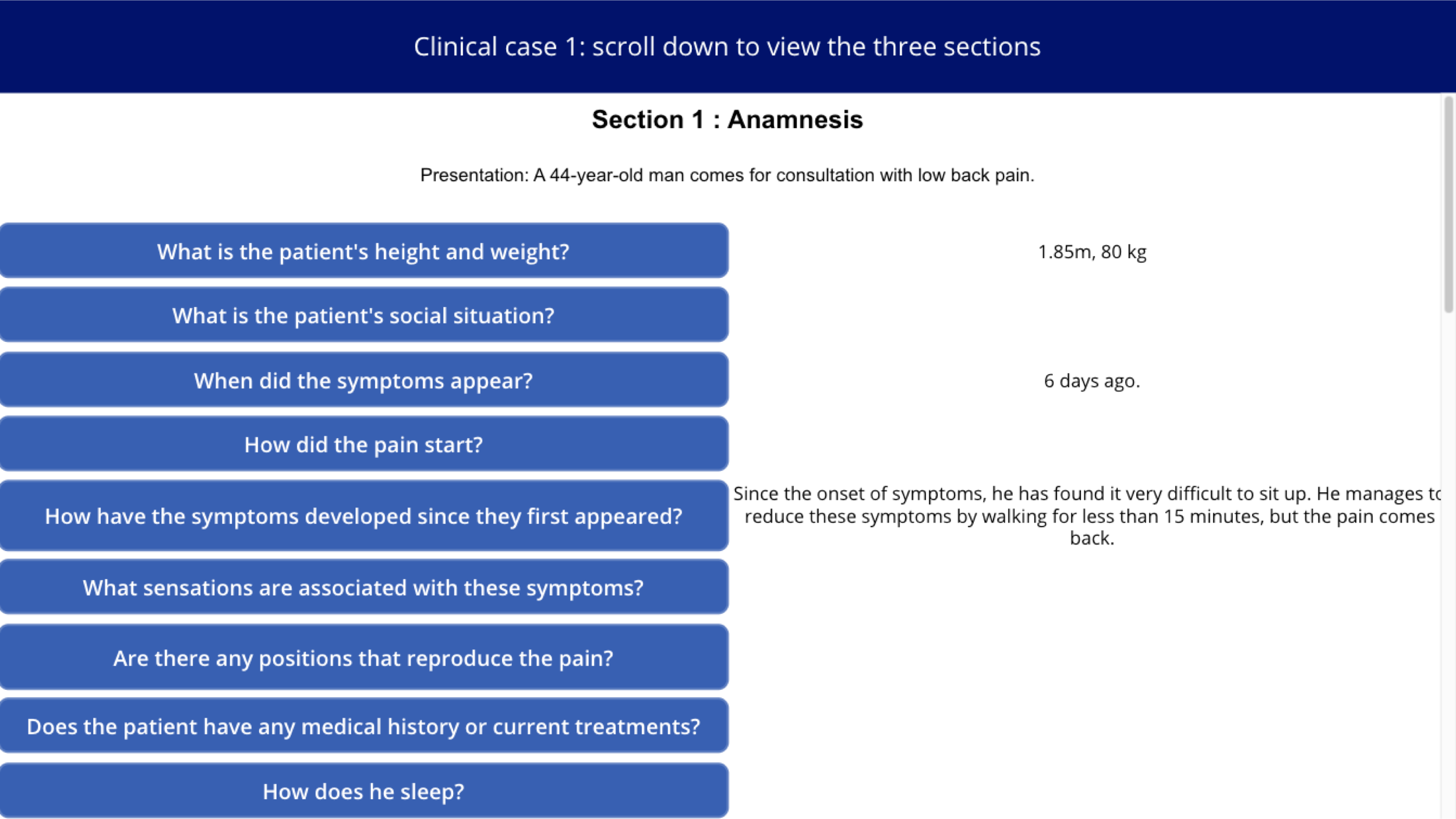

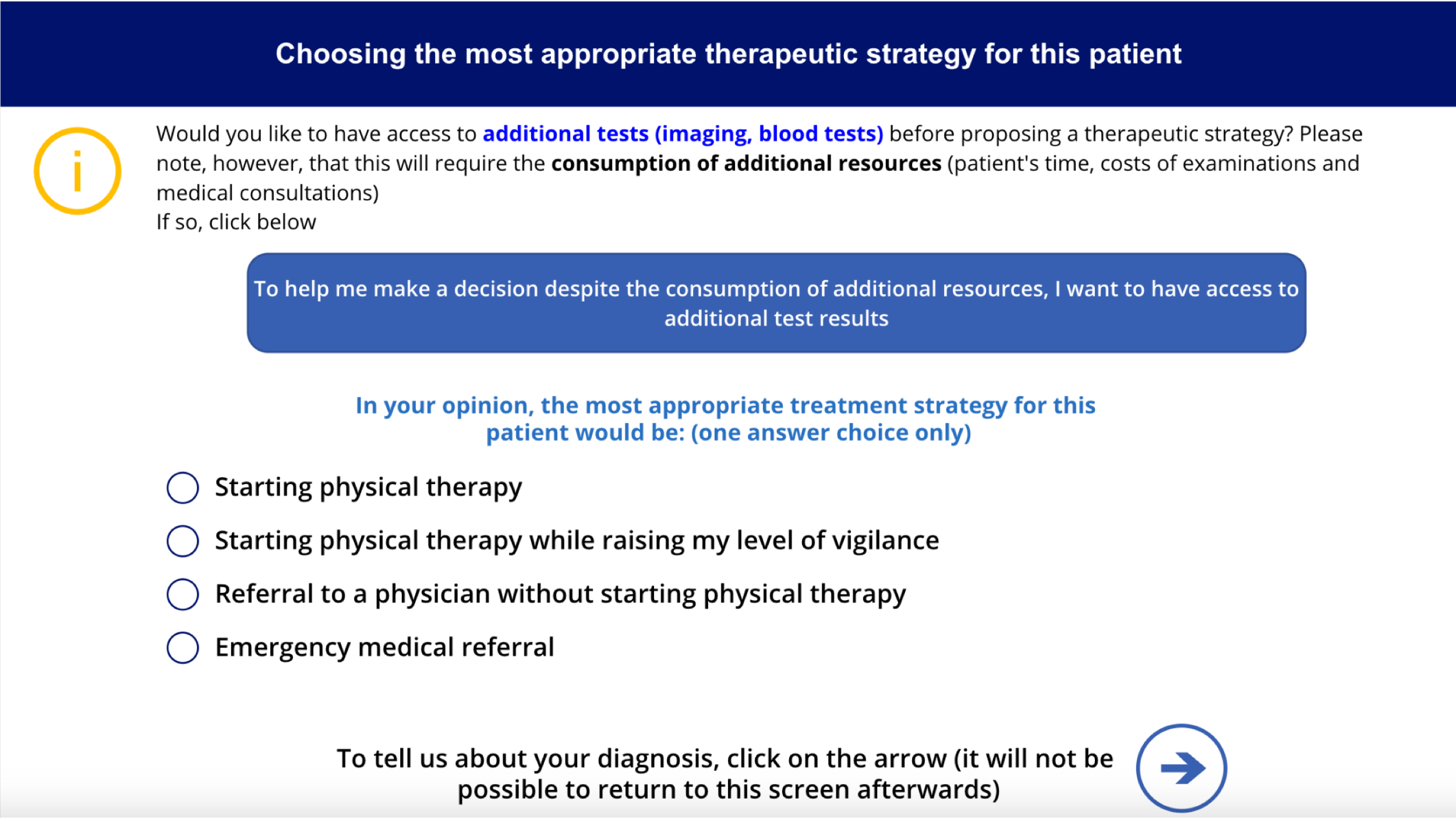

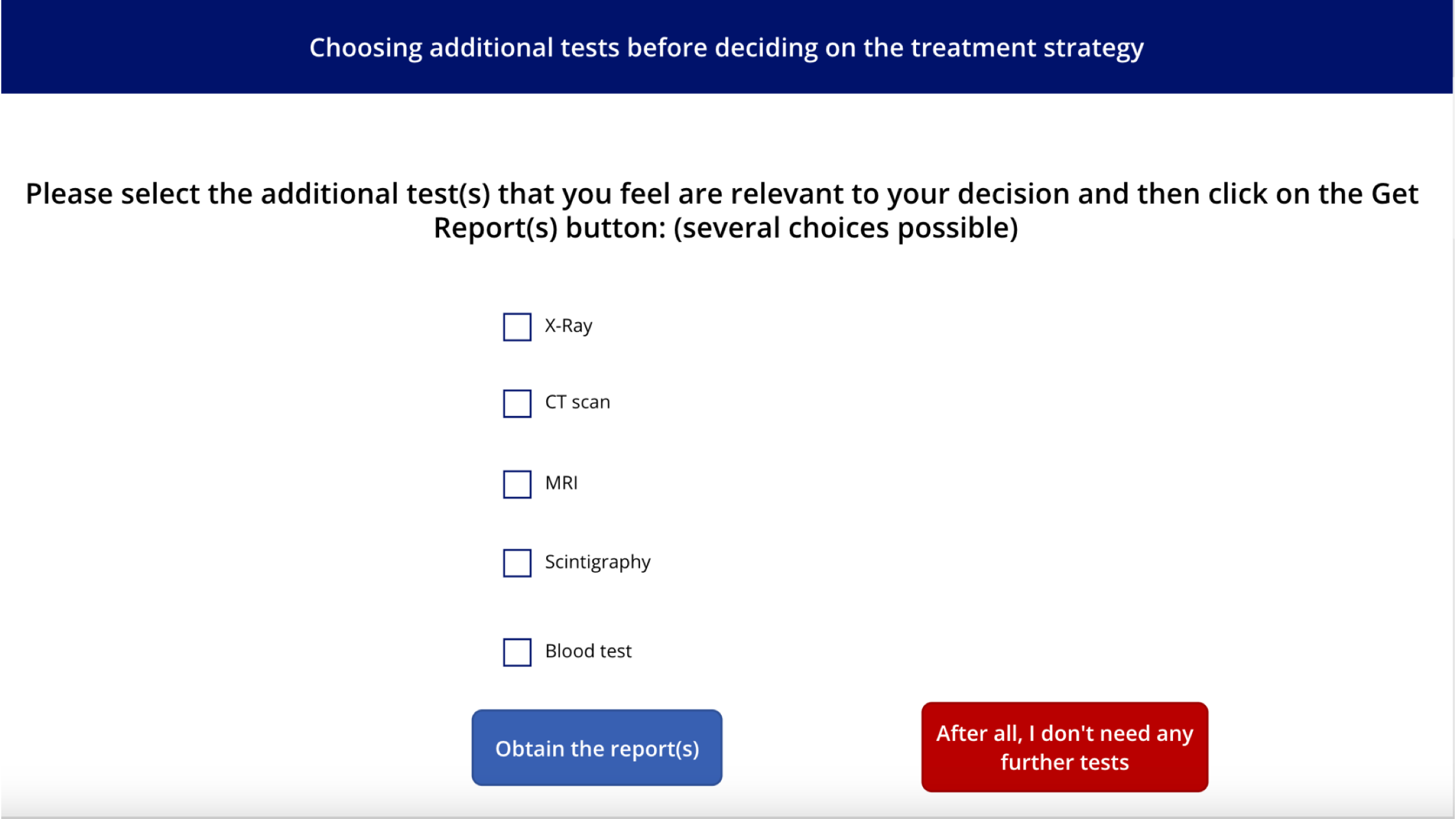

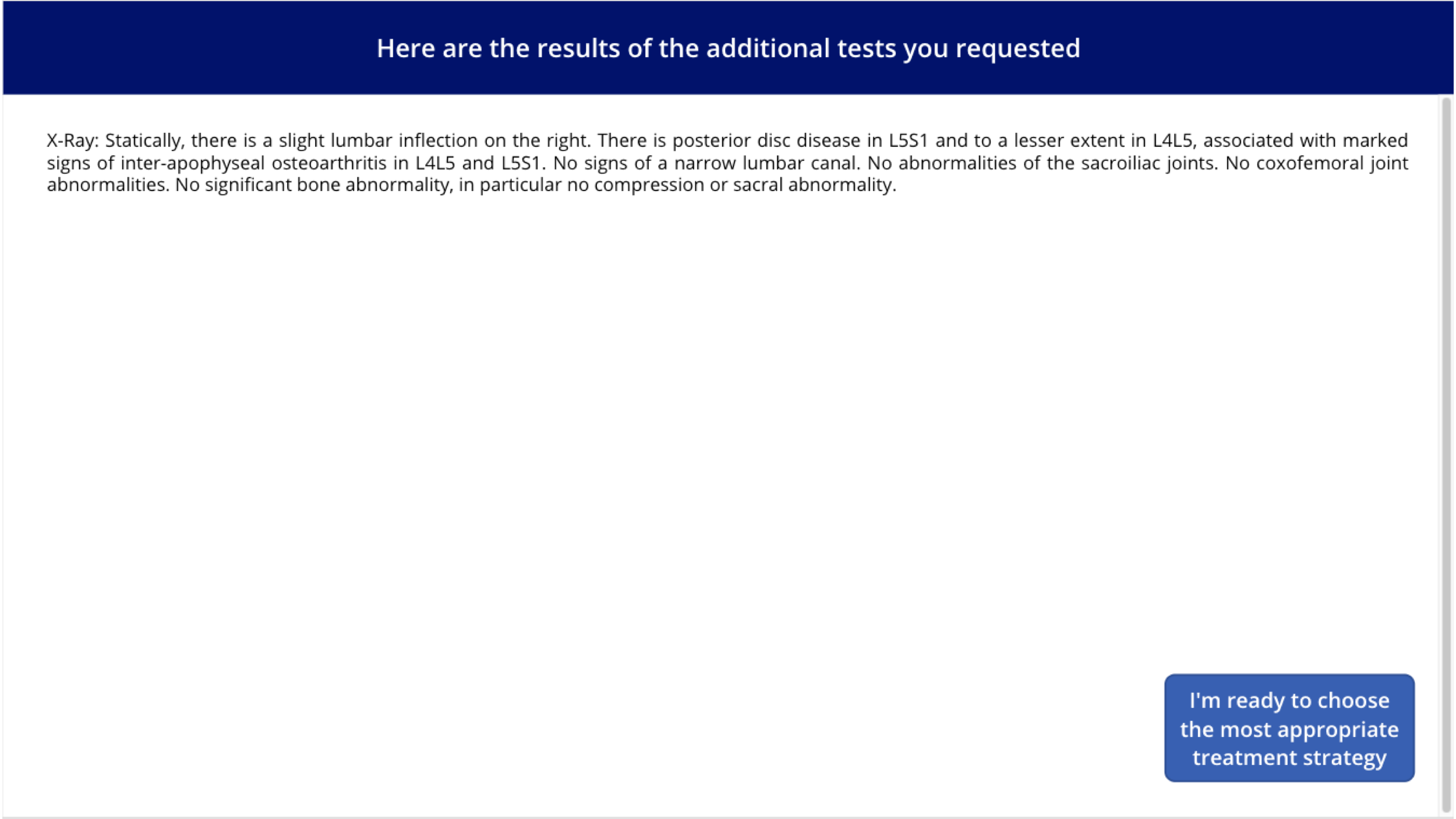

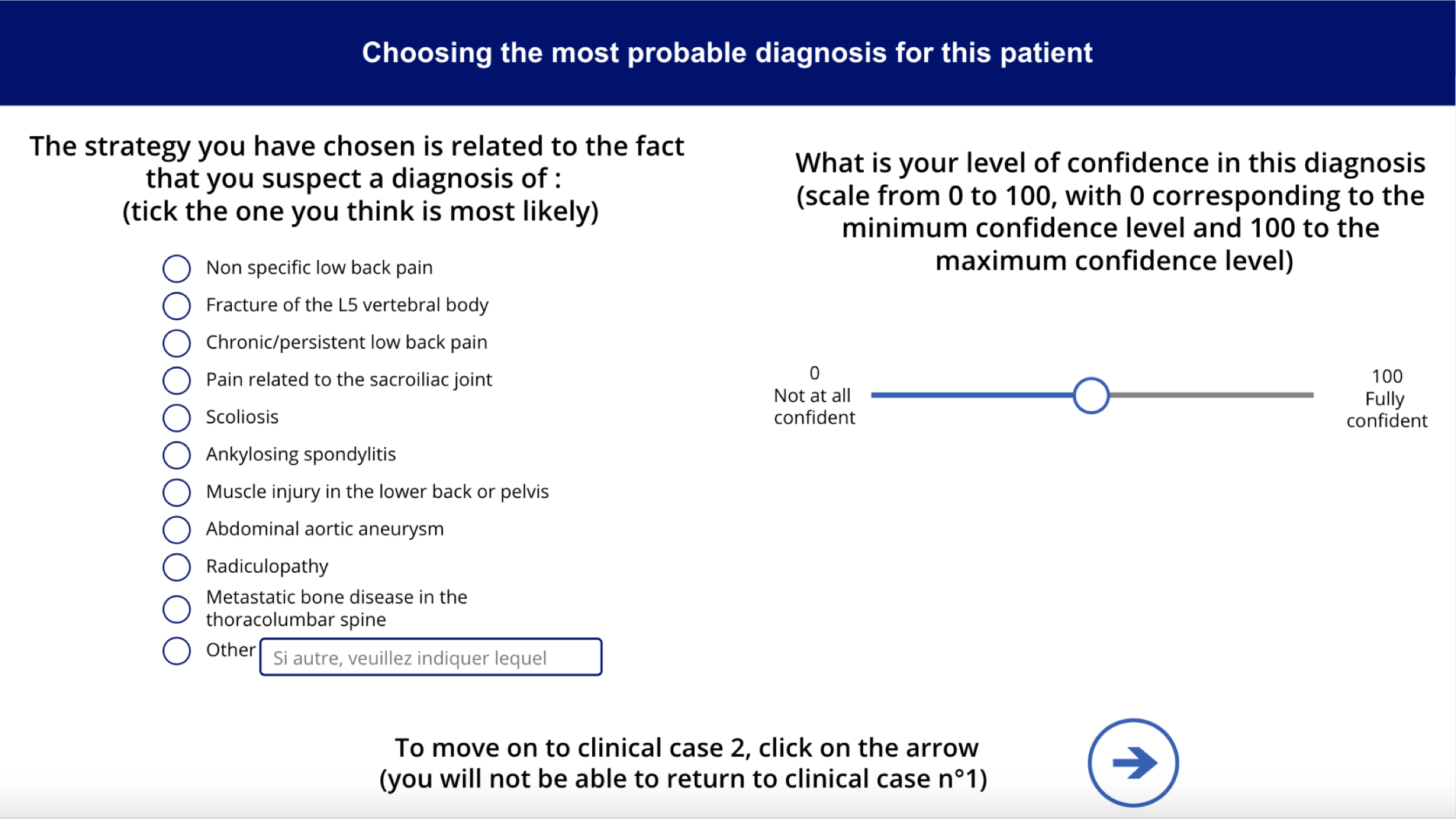

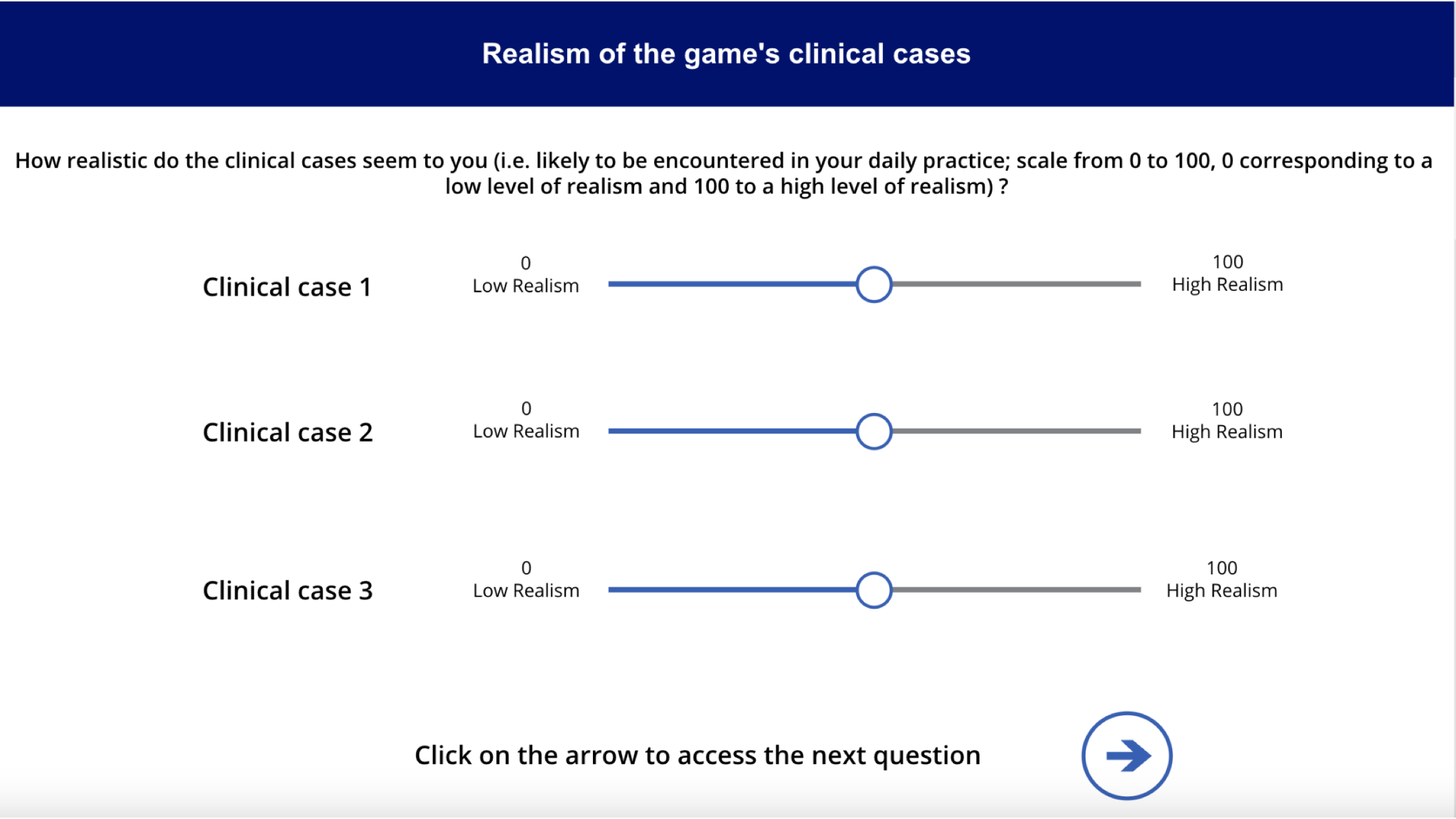

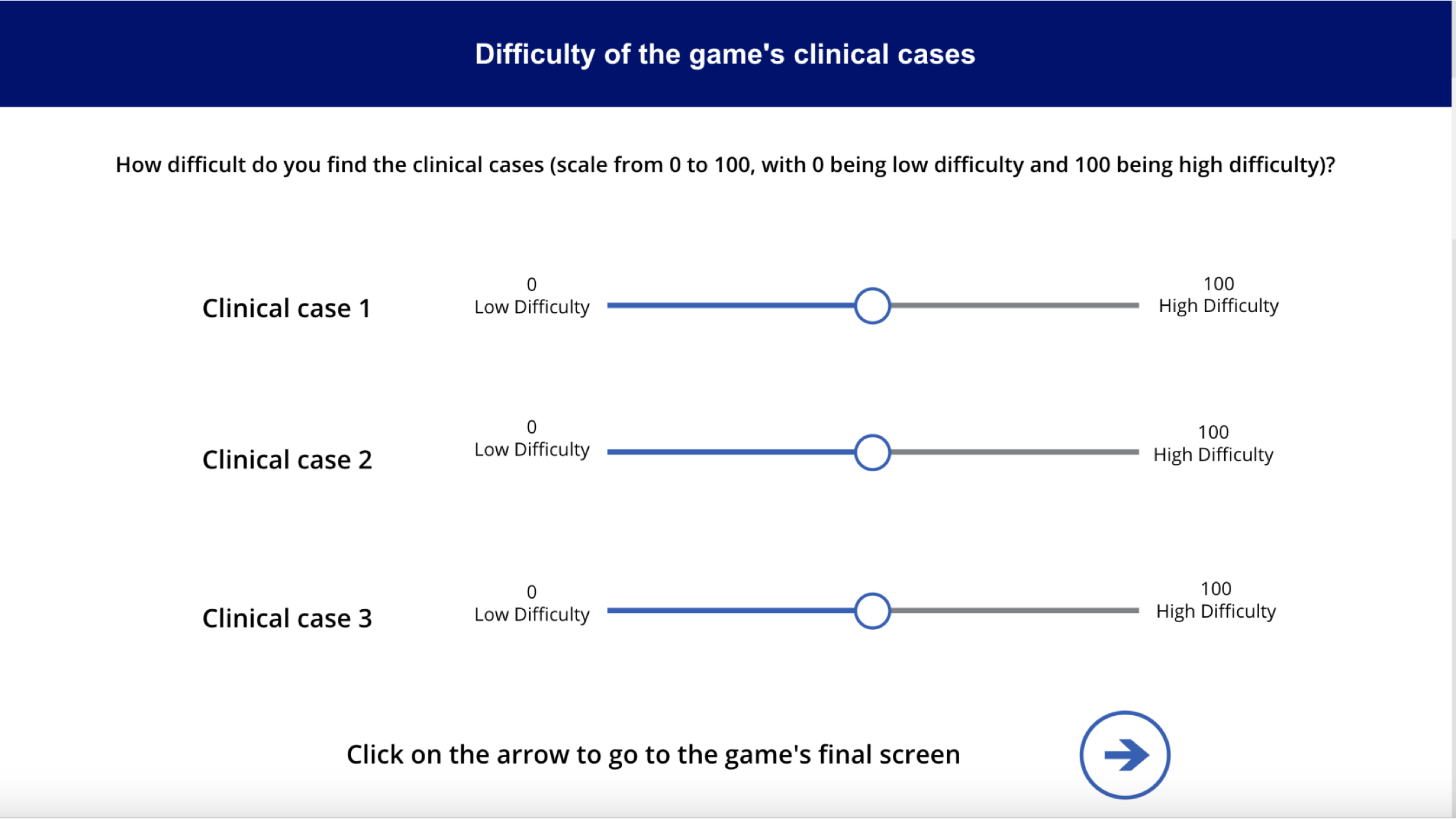

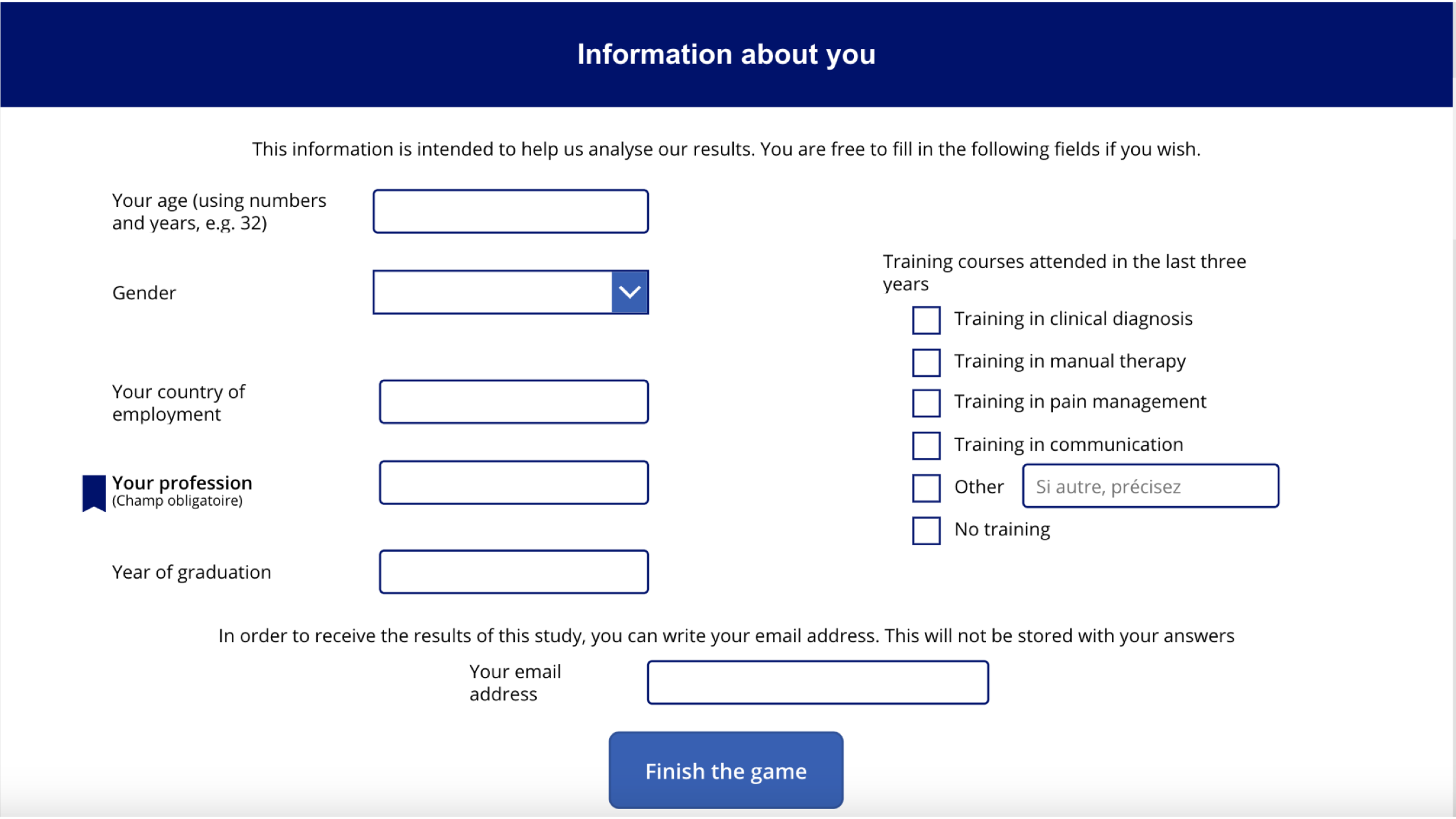

Supplement: Multimedia Appendix 1 [file rehab_v12i1e73818_app1.doc]
