# Supplementary material for: Effect of Intolerance of Uncertainty and Resource Consumption on Therapeutic Strategies Chosen by Physiotherapists: Virtual Patient Study
Source: JMIR Rehabil Assist Technol. 2025 Nov 18;12:e73818. doi: 10.2196/73818 (PMC12673305; doi:10.2196/73818)
Supplement: Multimedia Appendix 2 [file rehab_v12i1e73818_app2.docx]

# SUPPLEMENTARY MATERIAL

**Clinical case 1 (difficulty level : easy - suggestive of common non-specific lumbago)**

*Block 1 : History*

Presentation: A 44-year-old man comes for consultation with low back pain.

Question: What is the patient's height and weight?

Answer: 1.85 m, 80 kg

Question: What is the patient's social situation?

Answer: The patient is a trader, currently going through divorce proceedings with his ex-wife.

Question: When did the symptoms appear?

Answer: 6 days ago

Question: How did the pain start?

Answer: He woke up one morning with a pain in his back for no particular reason.

Question: How have the symptoms developed since they first appeared?

Answer: Since the onset of symptoms, he has found it very difficult to sit down. He manages to reduce these symptoms by walking for more than 15 minutes, but the pain soon returns, accompanied by a pain in his back.

Question: What sensations are associated with these symptoms?

Answer: The pain in his back bothers him in his daily activities.

Question: Are there any positions that reproduce the pain?

Answer: He is particularly uncomfortable sitting down. He says that he also has difficulty with everyday tasks such as putting on trousers and socks. If he does it too suddenly, it triggers severe back pain.

Question: Does the patient have any medical history or current treatments?

Answer: The patient has type 1 diabetes and ruptured his right anterior cruciate ligament 5 years ago.

Question: How does he sleep?

Answer: The patient tells you that he is bothered by pain at night, but that he still gets plenty of restful sleep.

Question: What are the patient's leisure activities?

Answer: He's not doing any physical activity at the moment, as he doesn't have the time because of his work schedule.

Question: What impact do the symptoms have on the patient?

Answer: He finds it difficult to concentrate at work. This handicaps him, especially as the atmosphere at work is not good. Recently, the company's results have not been good, which adds to the pressure from his superiors and the goals to be achieved.

Question: What are the patient's main objectives and expectations in terms of treatment?

Answer : To improve his situation, to be able to work without discomfort, to resume physical activity

*Block 2 : Physical examination*

Question: What visual observations do you make?

Answer: You look at the patient from the front and notice that he is standing relatively straight, with his left shoulder higher than his right. He also has varus knees. From the back, the left scapula is more protruding than the right, and the head is tilted more to the left. In profile, the lumbar lordosis is reduced. There are no skin or trophic signs (redness, scarring, swelling in the area of the patient's pain).

Question: Are there any identified nerve disorders?

Answer: No central or peripheral neurological disorders (large and small fibres) were identified.

Question: What are the main findings of the physical examination (non-exhaustive list)?

Answer :

Palpatory: sensitive lumbar spinal muscles, reproduce the patient's pain. Mechanosensitivity not reproduced by palpation.

Clinical tests:

- Gillet test: negative
- PKB (Prone Knee Bend test)/SKB (Slump Knee Bend test)/Léri's sign: negative
- Schober's test: +17 cm
- Fingertip-to-floor test: no discomfort, 22 cm distance from the ground
- Patrick's test (=FABER test): negative on both sides
- Slump test: negative
- Downing test (lower limb lengthening/shortening test): negative
- Cluster of Laslett: 0/5
- Lasègue test/SLR (Straight Leg Raise): negative
- DN4 (Douleur Neuropathique 4): negative
- No directional preference identified

Question: What are the results of the specific questionnaires carried out?

Answer

- Orëbro < 49 (low risk of future work disability)
- Start back tools: 3/9 (low risk of chronicity)
- FABQ (Fear-Avoidance Beliefs Questionnaire): 23/96 (the higher the score, the higher the beliefs and fears in relation to work and physical activity)
- Roland-Morris Disability Questionnaire: 6/24 (the higher the score, the greater the functional impact of low back pain)

*Block 3: Treatments used so far*

Question: What non-drug strategies have been used to ease his pain?

Answer: He applied a hot water bottle to the painful area, but it didn't really improve.

Question: What medication was used to relieve his pain?

Answer: He didn't take any medication.

Question: In view of all this, you decide to summarize the information you have gathered.

Answer: The patient goes on to thank you for understanding his problem, adding that he spends about 10 to 12 hours sitting down because of his work.

*Block 4: Imaging*

Question: Would you like an X-Ray?

Answer:

X-Ray: Mild right lumbar curve can be seen.. There is degenerative disc disease in L5S1 and to a lesser extent in L4L5, associated with severe degenerative facet joint arthrosis from L4L5 through L5S1. No evidence of lumbar spinal stenosis. No abnormalities of the sacroiliac joints. No hip joint abnormalities. No significant bone abnormality, in particular no compression or sacral abnormality.

Question: Would you like a CT scan?

Answer:

CT scan: Spinal canal dimensions are normal. No evidence of disc bulges from L3L4 to L4L5. Left facet joint osteoarthritis at the L4L5 level with inferior and superior facet hypertrophy with patent left intervertebral foramen. At the L5S1 level, there is a left posterolateral disc bulge without causing nerve root impingement. There were no abnormalities in the sacroiliac or hip joints.

Question: Would you like an MRI scan?

Answer:

MRI: The thecal sac and conus are normal. Vertebral body marrow signal and disc signal are normal. At L3L4 and L4L5 levels, moderate degenerative disc disease without disc protrusion. Mild Ligamentum flavum hypertrophy is seen at the L4L5 level. At the L5S1 level, diffuse intervertebral disc bulge with a mild central protrusion. Vertebral body degenerative changes seen on multiple levels, especially from L4L5 through L5D1. No central canal stenosis. The sacroiliac and hip joints are unremarkable.

Question: Would you like a scintigraphy?

Answer:

Scintigraphy: Scintigraphy with no particular abnormalities.

Question: Would you like a biological examination?

Answer:

- Blood test (Reference value = RV)
- Red blood cells 5.3 M/mm3 (RV 4.50-6.50)
- Haemoglobin 14.1 g/100 mL (RV 13.0-17.0)
- Haematocrit 44.7% (RV 40.0-54.0)
- White blood cells 8900 /mm3 (RV 4000-10000)
- Platelets 381000 /mm3 (RV 150000-500000)
- Erythrocyte Sedimentation rate 1st hour 1mm (RV<10)/ 2nd hour 2mm (RV<20)
- C-Reactive Protein (CRP) 2 mg/L (RV<6mg/L)
- Fasting blood glucose 0.82 g/L (RV 0.70-1.05)
- Creatinine 9.4 mg/L (RV 6.0-11.0)
- Calculation of estimated clearance (Cockroft and Gault formula) 113mL/min
- No HLA B27 marker

**clinical case 2 (difficulty level : medium - suggestive of ankylosing spondylitis)**

*Block 1 : History*

Presentation: A 27-year-old man comes for consultation with low back pain.

Question: What is the patient's height and weight?

Answer: 1.75m, 68.5 kg

Question: What is the patient's social situation?

Answer: He lives with his partner. He is in his final year of a doctorate in biochemistry.

Question: When did the symptoms appear?

Answer: This episode occurred 5 days ago, but he has had similar pain several times before.

Question: How did the pain start?

Answer: The patient does not know what could have caused the pain.

Question: How have the symptoms changed since they first appeared?

Answer: The patient has not noticed any significant change since the onset of the pain.

Question: What sensations are associated with these symptoms?

Answer: When these symptoms appear, he is usually able to maintain his physical activities except during episodes of excessive pain. The patient has noticed that the onset of his painful periods is associated with stress due to professional constraints and/or excessive sporting activity. Finally, he tells you about another pain in his left heel that can sometimes bother him when running.

Question: Are there any positions that reproduce the pain?

Answer: Prolonged sitting.

Question: Does the patient have a history of the condition and any ongoing treatment?

Answer: The patient's history includes a right ankle sprain in 2017, a fractured right shoulder following a scooter fall in 2012 and psoriasis diagnosed in 2020. He is not taking any medication.

Question: How is his sleep?

Answer: He has noticed that his sleep is often altered during painful episodes and that he finds it difficult to go back to sleep once he has woken up.

Question: What are the patient's leisure activities?

Answer: He does a lot of physical activity: running, crossfit (3 to 5 times a week).

Question: What impact do the symptoms have on the patient?

Answer: He says that he has often had periods of lower back pain since he was 20. During periods of severe pain, he feels rusty, especially in the morning. He manages to live with it, but it worries him when it deprives him of activities that bring him well-being, as is currently the case. He insists on the importance of maintaining an acceptable level of physical activity and pain, now that his thesis is due.

Question: What are the patient's main objectives and expectations in terms of treatment?

Answer : To resume all physical activities as before, to put an end to the discomfort he has suffered for many years, to be helped with his pain and to find strategies for managing their back pain

*Block 2 : Physical examination*

Question: What visual observations do you make?

Answer: You look at the patient from the front and notice that he is standing with his shoulders rolled up and that his right shoulder is higher than his left. He also has valgus knees. From the back, you can see that the spine is not aligned with the vertical but deviates to the left, with the head leaning more to the left. In profile, the thoracic kyphosis is more marked and the lumbar spine is straight. There are no skin or trophic signs (redness, scarring, swelling in the area of the patient's pain).

Question: Are there any identified nerve disorders?

Answer: No central or peripheral neurological disorders (large and small fibres) were identified.

Question: What are the main findings of the physical examination (non-exhaustive list)?

Answer:

Palpatory: sensory lumbar spines, psoas, piriformis and quadratus lumborum do not reproduce the patient's pain. Mechanosensitivity not reproduced by palpation.

Clinical tests:

- Gillet test: Positive at G (the left Posterior Superior Iliac Spine ascends at the end of flexion).
- PKB (Prone Knee Bend test)/SKB (Slump Knee Bend test)/Léri's sign: negative
- Schober test: 12cm
- Fingertip-to-floor test: reproduces pain, 18cm distance from the ground
- Patrick's test (=FABER): positive at G
- Slump test: negative
- Downing test (lower limb lengthening/shortening test): positive at D(anterior ilium)
- Cluster of Laslett: 3/5
- Lasègue test/SLR (Straight Leg Raise): negative
- DN4 (Douleur Neuropathique 4): negative
- No directional preference identified

Question: What are the results of the specific questionnaires carried out?

Answer:

- Orëbro > 49 (higher risk of future work disability).
- Start back tools: 4/9 and sub questionnaire < 4 (average risk of chronicity)
- FABQ (Fear-Avoidance Beliefs Questionnaire): 16/96 (the higher the score, the higher the beliefs and fears in relation to work and physical activity)
- Roland-Morris Disability Questionnaire: 5/24 (the higher the score, the greater the functional impact of low back pain)

*Block 3: Treatments used so far*

Question: What non-drug strategies have been used to ease his pain?

Answer: He limits physical activity when the pain is severe. When it's bearable, he tries to go for a walk, which seems to do him good.

Question: What drugs have been used to relieve his pain?

Answer: He says that during painful episodes he is prescribed non-steroidal anti-inflammatory drugs (NSAIDs), which give him relief but cause him stomach pains. He would like to stop taking NSAIDs.

Question: In view of all this, you decide to summarise the information you have gathered.

Answer: The patient goes on to confirm that you have understood his problem and sees nothing more to add.

*Block 4 : Imaging*

Question: Would you like an X-Ray?

Answer:

X-Ray: A pelvic obliquity on the left is seen in static position.. Examination reveals mainly multilevel degenerative disc disease with pinching lateral to the left at L3-L4, pinching lateral to the right at the L4-L5 level and the L5-S1 level. Presence of sacroiliitis. No hip joint abnormalities. No significant bone abnormality, in particular no compression or sacral abnormality.

Question: Would you like a CT scan?

Answer:

CT scan: No lumbar static abnormalities. No transitional anomalies. No lytic or condensing bone lesions. No evidence for degenerative changes of the vertebral bodies or facet joints osteoarthritis.. Spinal canal dimensions are normal. No spontaneously detectable paravertebral soft tissue abnormalities. Medial and left paramedian disc protrusion, filling the anterior epidural fat and exerting a mass effect on the thecal sac and the homolateral L3 nerve root at the level of its emergence. Right disc protrusion at the L4L5 level without impingement. Discrete paramedian disc protrusion at the L5S1 level. Sacroilitis is identified on the CT scan.

Question: Would you like an MRI scan?

Answer:

MRI: Degenerative disc disease is seen in the last three lumbar levels with disk space narrowing and posterior disc protrusion. At L3-L4 level: there is a left posterolateral disc protrusion in contact with the dural sac. At L4-L5 level: mild disc protrusion lateralized on the right side. At L5-S1: overall disc bulge with a medial disc protrusion. No clear discovertebral anomaly of the dorsal spine. No spinal cord abnormality. No suspicious bony abnormality. No facet joints anomaly. No significant central canal stenosis. No significant foraminal stenosis. Pseudo widening of the sacroiliac joint space with bone oedema in T2 sequence suggestive of sacroiliitis. The hip joints are intact.

Question: Would you like a scintigraphy?

Answer: Scintigraphy with no particular abnormalities.

Question: Would you like a biological examination?

Answer: Blood test (Reference value = RV)

- Red blood cells 5.1 M/mm3 (RV 4.50-6.50)
- Haemoglobin 13.9 g/100 mL (RV 13.0-17.0)
- Haematocrit 47.4% (RV 40.0-54.0)
- Leukocytes 8800 /mm3 (RV 4000-10000)
- Platelets 302000 /mm3 (RV 150000-500000)
- Sedimentation rate 1st hour 8mm (RV<10)/ 2nd hour 19mm (RV<20)
- C-Reactive Protein (CRP) 15 mg/L (RV<6mg/L)
- Fasting blood glucose 1.01g/L (RV 0.70-1.05)
- Creatinine 9.7 mg/L (RV 7.0-13.0)
- Calculation of estimated clearance (Cockroft and Gault formula) 111 mL/min
- No HLA B27 marker

**clinical case 3 (difficulty level : difficult - suggestive of metastatic bone disease in the thoracolumbar spine)**

*Block 1 : History*

Presentation: A 54-year-old woman comes for consultation with low back pain.

Question: What is the patient's height and weight?

Answer: 1.62 m, 58.5 kg

Question: What is the patient's social situation?

Answer: She is married and lives in a first floor flat. She is a secondary school teacher.

Question: When did the symptoms first appear?

Answer: Her pain started 3 or 4 weeks ago.

Question: How did the pain start?

Answer: She can't think of any particular reason, perhaps when she helped her husband unload the boot on returning from a trip.

Question: How have the symptoms developed since they first appeared?

Answer: From the beginning, the pain has gradually increased.

Question: What sensations are associated with these symptoms?

Answer: She shows you the middle and lower back. She says it goes down behind her right leg. She has also had difficulty walking since the pain increased.

Question: Are there any positions that reproduce the pain?

Answer: Nothing of note.

Question: Does the patient have any history of pain or ongoing treatment?

Answer: She remembers breaking her wrist several years ago. She has been taking Levothyrox daily since her thyroid cancer 3 years ago (the thyroid has since been surgically removed).

Question: How does she sleep?

Answer: The patient tells you that her nights are very complicated at the moment, she's been sleeping less since the pain appeared. She finds her sleep less restorative.

Question: What leisure activities does the patient do?

Answer: She goes Nordic walking every Sunday and does some painting.

Question: What impact do the symptoms have on the patient?

Answer: She does fewer things than before. She tried to resume her daily activities (Nordic walking, painting) 15 days ago, but stopped after a few days because she couldn't see any improvement. She's really worried and dreads having a pulled or torn muscle.

Question: What are the patient's main objectives and expectations in terms of treatment?

Answer: She doesn't quite understand your question, the doctor told her that with physiotherapy it would go away, simply no more pain, do what is written on the prescription, it was her doctor who told her to go and see a physiotherapist.

*Block 2 : Physical examination*

Question: What visual observations do you make?

Answer: You look at the patient from the front and notice that she is standing with her shoulders back and that her shoulders are at the same height. In addition, her knees are valgum/varum free. From the back, there is no deviation of the spine. In profile, there is no increase in kyphosis or lordosis. There are no dermatological signs (redness, scarring, swelling in the area of the patient's pain).

Question: Have any nervous disorders been identified?

Answer: No central or peripheral neurological disorders (large and small fibres) were identified.

Question: What are the main findings of the physical examination (non-exhaustive list)?

Answer:

Palpatory: lumbar spinal tendons, psoas, piriformis and quadratus lumborum reproduce the patient's pain. Mechanosensitivity not reproduced by palpation.

Clinical tests:

- Gillet test: Positive at G (the left Posterior Superior Iliac Spine ascends at the end of flexion).
- PKB (Prone Knee Bend test)/SKB (Slump Knee Bend test)/Léri's sign: negative
- Schober's test: 13cm
- Fingertip-to-floor test: no discomfort, 25cm distance from the ground
- Patrick's test (=FABER test): positive at R and L
- Slump test: negative
- Downing test (lower limb lengthening/shortening test): short.
- Cluster of Laslett: 1/5
- Lasègue test/SLR (Straight Leg Raise): negative
- DN4 (Douleur Neuropathique 4): negative
- No directional preference identified

Question: What are the results of the specific questionnaires carried out?

Answer:

- Orëbro > 49 (higher risk of future work disability).
- Start back tools: 5/9 and sub questionnaire > 4 (high risk of chronicity)
- FABQ (Fear-Avoidance Beliefs Questionnaire): 17/96 (the higher the score, the higher the beliefs and fears in relation to work and physical activity)
- Roland-Morris Disability Questionnaire: 10/24 (the higher the score, the greater the functional impact of low back pain)

*Block 3: Treatments used so far*

Question: What non-drug strategies have been used to ease her pain?

Answer: Her husband offered to massage her, which felt very good at the time but didn't last over time.

Question: What medication was used to ease her pain?

Answer: She tells you that she's not a fan of medication, that she took a medicine for a week, can't remember the exact name, but it contained codeine. In any case, it hadn't helped her much.

Question: In view of all this, you decide to summarise the information you have gathered.

Answer: After the summary, the patient thinks she has forgotten to give you some information. She notes that the pain is worse at night. She says that taking the codeine-based medication has increased her stomach pains. She called her doctor to check whether it was the dosage of Levothyrox that was causing her stomach pains, as she had had the same pains at the start of treatment. However, she has no problems with bowel movements, as she had at the time.

*Block 4 : Imaging*

Question: Would you like an X-Ray?

Answer:

X-Ray: Normal static front and side views. No significant pelvic tilt. Posterior degenerative disc disease at L5S1 level. No abnormal bone structure. Signs of incipient facet joints osteoarthritis. No evidence of lumbar spinal stenosis. Normal appearance of sacroiliac and hip joints.

Question: Would you like a CT scan?

Answer:

CT scan: No abnormalities of lumbar static views. No transitional abnormalities. Lumbar canal of normal calibre. Global paramedian disc bulge at L5S1 level. Signs of bone metastases in the vertebral bodies on several dorsolumbar levels, in relation to the patient's history of neoplasia. Slight facet joints osteoarthritic changes. No abnormalities in the sacroiliac or hip joints.

Question: Would you like an MRI scan?

Answer:

MRI: Degenerative disc disease in the last two lumbar levels with disk space narrowing and posterior disc bulges. At the L4-L5 level: discrete overall disc protrusion. At the L5-S1 level: medial disc protrusion. No significant central canal stenosis. No significant foraminal stenosis. MRI showed several focal lesions of different sizes in the vertebral bodies on several dorsolumbar levels: they appeared in high signal intensity on T1-weighted images and on T2-weighted images in favour of bone metastases. No signs of extension to adjacent soft tissues. Integrity of vasculo-nervous structures. No visible abnormalities in the sacroiliac joints. Integrity of the hip joints.

Question: Would you like a scintigraphy?

Answer:

Scintigraphy: Multiple areas of hyperfixation in the axial skeleton. The entire pelvic complex seems to be spared.

Question: Would you like a biological examination?

Answer:

- Blood test (reference value = RV) :
- Red blood cells 4.8 M/mm3 (RV 4.50-6.50)
- Haemoglobin 12.1 g/100 mL (RV 12.0-16.0)
- Haematocrit 42.4% (RV 40.0-54.0)
- Leukocytes 9100 /mm3 (RV 4000-10000)
- Platelets 358000 /mm3 (RV150000-500000)
- Sedimentation rate 1st hour 7mm (RV<10)/ 2nd hour 19mm (RV<20)
- C-Reactive Protein (CRP) 15 -20 mg/L (RV<6mg/L)
- Fasting blood glucose 0.78g/L (RV 0.70-1.05)
- Creatinine 10.1 mg/L (RV 6.0-11.0)
- Calculation of estimated clearance (Cockroft and Gault formula) 59mL/min
- No HLA B27 marker
